# Second-line Eribulin in Triple Negative Metastatic Breast Cancer patients. Multicentre Retrospective Study: The TETRIS Trial

**DOI:** 10.7150/ijms.54996

**Published:** 2021-03-27

**Authors:** Eriseld Krasniqi, Laura Pizzuti, Maria Rosaria Valerio, Elisabetta Capomolla, Claudio Botti, Giuseppe Sanguineti, Paolo Marchetti, Elisabetta Anselmi, Silverio Tomao, Antonio Giordano, Corrado Ficorella, Katia Cannita, Lorenzo Livi, Icro Meattini, Maria Mauri, Filippo Greco, Enzo Maria Veltri, Andrea Michelotti, Luca Moscetti, Francesco Giotta, Vito Lorusso, Ida Paris, Federica Tomao, Daniele Santini, Giuseppe Tonini, Alice Villa, Vittorio Gebbia, Teresa Gamucci, Gennaro Ciliberto, Isabella Sperduti, Marco Mazzotta, Maddalena Barba, Patrizia Vici

**Affiliations:** 1 Division of Medical Oncology 2, IRCCS Regina Elena National Cancer Institute, Rome, Italy.; 2 Department of Surgical, Oncological and Oral Sciences, Medical Oncology Unit, University of Palermo, Italy.; 3 Department of Surgery, IRCCS Regina Elena National Cancer Institute, Rome, Italy.; 4 Department of Radiation Oncology, IRCCS Regina Elena National Cancer Institute, Rome, Italy.; 5 Medical Oncology Unit B, Policlinico Umberto I, Rome, Italy.; 6 Medical Oncology Unit, Azienda Ospedaliera Universitaria Sant'Andrea, Rome, Italy.; 7 Department of Radiological, Oncological and Anatomo-Pathological Sciences, 'Sapienza' University of Rome, Policlinico Umberto I, Rome, Italy.; 8 Sbarro Institute for Cancer Research and Molecular Medicine and Center of Biotechnology, College of Science and Technology, Temple University, Philadelphia, Pennsylvania, USA.; 9 Medical Oncology, Department of Biotechnological and Applied Clinical Sciences, University of L'Aquila, L'Aquila, Italy.; 10 Medical Oncology, St. Salvatore Hospital, L'Aquila, Italy; 11 Radiation Oncology Unit and Department of Clinical and Experimental Biomedical Sciences “Mario Serio”, Azienda Ospedaliera Universitaria Careggi, University of Florence, Florence, Italy.; 12 Division of Oncology, San Giovanni Addolorata Hospital, Rome, Italy.; 13 Department of Pathology, Surgery and Oncology, “Mater Salutis” Hospital, ULSS21, Verona, Italy.; 14 Oncology Unit, S. Maria Goretti Hospital, Latina, Italy.; 15 UO Oncologia Medica I, S. Chiara Hospital, Dipartimento di Oncologia, Dei Trapianti e Delle Nuove Tecnologie, Azienda Ospedaliera Universitaria Pisana, Pisa, Italy.; 16 Division of Medical Oncology, Department of Oncology and Hematology, University Hospital of Modena, Modena, Italy.; 17 Department of Medical Oncology, “Giovanni Paolo II” Institute, Bari, Italy.; 18 Gynecology Oncology Unit, Catholic University of the Sacred Heart, Rome, Italy.; 19 Department of Gynecology-Obstetrics and Urology, “Sapienza” University of Rome, Rome, Italy.; 20 Department of Oncology, University Campus Biomedico of Rome, Rome, Italy.; 21 Department of Medical Oncology, Policlinico Universitario “A. Gemelli”, Rome, Italy.; 22 Medical Oncology, La Maddalena Nursing Home, University of Palermo, Palermo, Italy; 23 Medical Oncology Unit, Sandro Pertini Hospital, Rome, Italy.; 24 Scientific Direction, IRCCS Regina Elena National Cancer Institute, Rome, Italy.; 25 Bio-Statistics Unit, IRCCS Regina Elena National Cancer Institute, Rome, Italy.

**Keywords:** eribulin mesylate, triple negative metastatic breast cancer, efficacy outcomes, toxicity outcomes, chemotherapy

## Abstract

**Introduction:** Large and consistent evidence supports the use of eribulin mesylate in clinical practice in third or later line treatment of metastatic triple negative breast cancer (mTNBC). Conversely, there is paucity of data on eribulin efficacy in second line treatment.

**Methods:** We investigated outcomes of 44 mTNBC patients treated from 2013 through 2019 with second line eribulin mesylate in a multicentre retrospective study involving 14 Italian oncologic centres.

**Results:** Median age was 51 years, with 11.4% of these patients being metastatic at diagnosis. Median overall survival (OS) and progression free survival (PFS) from eribulin starting were 11.9 (95%CI: 8.4-15.5) and 3.5 months (95%CI: 1.7-5.3), respectively. We observed 8 (18.2%) partial responses and 10 (22.7%) patients had stable disease as best response. A longer PFS on previous first line treatment predicted a better OS (HR=0.87, 95%CI: 0.77-0.99, p= 0.038) and a longer PFS on eribulin treatment (HR=0.92, 95%CI: 0.85-0.98, p=0.018). Progression free survival to eribulin was also favorably influenced by prior adjuvant chemotherapy (HR=0.44, 95%CI: 0.22-0.88, p=0.02). Eribulin was generally well tolerated, with grade 3-4 adverse events being recorded in 15.9% of patients.

**Conclusions:** The outcomes described for our cohort are consistent with those reported in the pivotal Study301 and subsequent observational studies. Further data from adequately-sized, ad hoc trials on eribulin use in second line for mTNBC are warranted to confirm our findings.

## Introduction

Approximately 15% of breast cancers are classified as triple-negative (TNBC), a subtype associated with aggressive clinical behavior and poor prognosis. Metastases in TNBC are described in about one-third of patients, with either recurrent or de novo metastatic disease 1.

Chemotherapy has long been considered the only active treatment for metastatic TNBC 2. This latter scenario has recently changed with the advent of polyadenosine diphosphate-ribosepolymerase inhibitors (PARPis) for patients harboring BRCA mutations 3. In addition, the contrasting evidence on the combination of chemotherapy and atezolizumab in patients with PD-L1 positive tumors intensely animates the scientific debate, due to the recently presented results from the IMpassion 130 and 131 trial 4,5.

Eribulin mesylate is a synthetic halichondrin B analog that inhibits the microtubule growth phase 6. Among its non-mitotic mechanisms of action, it is worth mentioning its anti-angiogenetic effects and its ability to reverse epithelial-mesenchymal transition process 7,8. Moreover, it was recently suggested an association between eribulin treatment and an increase in tumor-infiltrating lymphocytes (TILs), a relevant predictive and prognostic marker in triple negative (TN) disease 9. Eribulin is approved for treatment of patients with advanced breast cancer who are refractory to other treatments. This is based on the results from the EMBRACE trial, wherein eribulin was compared to treatment of physician choice, and from the Study 301, which evaluated eribulin in comparison with capecitabine 10,11. In specific regard to eribulin activity in TNBC, a pooled analysis including patients of the EMBRACE and 301 trials showed a survival benefit for patients receiving eribulin versus control or capecitabine 12. However, only the 301 Study enrolled TNBC patients receiving eribulin in second-line 13.

Herein we present our work aimed at investigating second-line eribulin efficacy in mTNBC in a historical cohort of patients treated at 14 Italian cancer centres.

## Methods

The TETRIS trial is a multicenter retrospective study which was designed to assess the efficacy of eribulin as second line of treatment in patients affected by mTNBC. The study was conducted in full accordance with the guidelines for Good Clinical Practice and the Declaration of Helsinki, and was approved by the institutional ethics committees of each center. Overall, 14 cancer centers adhered to our study. Written informed consent was obtained from all patients who remained alive at the time of trial approval. Patients were deemed suitable for inclusion in the TETRIS trial if diagnosed with mTNBC, and having received at least one cycle of eribulin (1.23 mg/m^2^) following failure of a first line chemotherapy. Eribulin had to be delivered between January 2013 and September 2019, since a minimum 12-month follow up was required. In addition, data availability was required concerning key patient- and disease clinical-pathological variables, along with treatment outcomes. Eribulin treatment was delivered until disease progression, unacceptable toxicity or patient refusal, and efficacy was evaluated according to Response Evaluation Criteria in Solid Tumors (v. 1.1). Adverse events were recorded and graded according to the National Cancer Institute Common Terminology Criteria for Adverse Events (v.4.0).

The primary objectives of the study were progression free survival (PFS), and overall survival (OS). Secondary endpoints included objective response and safety outcomes. Explorative analyses for potential clinical-pathological predictors of efficacy were also performed.

Descriptive statistics were used to characterize the study sample. Performance status was assessed according to the Eastern Cooperative Oncology Group (ECOG PS) prior to and following eribulin treatment and compared by Wilcoxon test. The Kaplan-Meier method and log-rank test were used to estimate survival and compare the inherent data across subgroups defined upon clinically and molecularly relevant variables. Univariate and multivariate Cox proportional hazards regression models were developed/built to evaluate associations of clinical-pathologic features with PFS and OS. Multivariate analysis was carried out including only variables testing significant in univariate analysis. The SPSS software (SPSS version 21.0, SPSS Inc., Chicago, IL) was used for all statistical evaluations. The significance levels for all performed tests was set at p<0.05.

## Results

**Figure 1:**
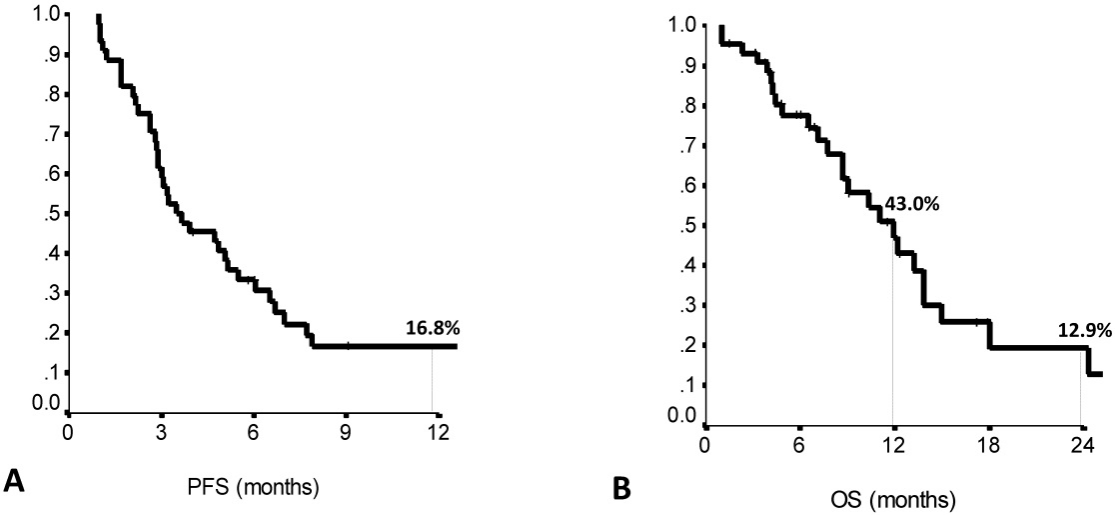
Second line eribulin treatment: 12-months PFS (A) and 12- and 24-months OS (B). PFS: Progression Free Survival. OS: Overall Survival. N: 44 patients.

Forty-four patients met the study inclusion criteria. Main patient and tumor characteristics are shown in Table 1. Median PFS to the previous first line treatment was 7.0 months (range: 1.0-21.0), as calculated by the Kaplan Meier product limit (Suppl Table S1). The main clinical outcomes of eribulin treatment are listed in Suppl Table S2. Median PFS on second line eribulin was 3.5 months (range: 1.7-5.3), with a one year-PFS rate of 16.7%. Median OS was 11.9 months (range: 8.4-15.5), with OS rates at 1-year and 2-years of 43.0% and 12.7%, respectively (Figure 1A and 1B). Second line eribulin did not yield any complete response (CR). However, 18.2% of partial responses (PR) and 22.7% of stable diseases (SD) were recorded.

**Figure 2:**
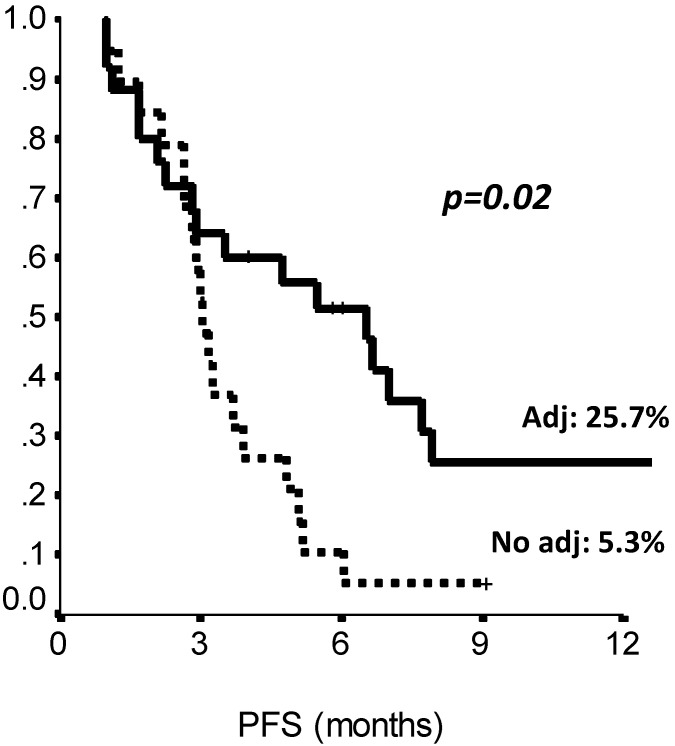
PFS by prior adjuvant treatment (Chemotherapy: yes vs no). Adj.: adjuvant, PFS: Progression Free Survival. N: 44 patients.

The comparison of survival curves showed that having received a previous adjuvant treatment predicted a better PFS on second line eribulin with respect to not having received it (p=0.02, log-rank test) (Fig. 2). Also, PFS on first line treatment impacted PFS to second line eribulin. As shown in Suppl Figure 1, the survival curve for PFS on eribulin was more favorable for those patients who had experienced a PFS longer than 10 months on the previous line of treatment, with PFS rates on eribulin at 12 months of 43.0% vs. 9.8% (p=0.03, log-rank test). Interestingly, difference in ECOG PS at eribulin starting, namely, 0 compared to 1-2, did not influence survival (p= 0.33, log-rank test). Univariate analysis (Suppl Table S3) confirmed that only adjuvant chemotherapy and first line PFS had a significant effect on eribulin PFS. Adjuvant treatment and PFS on first line maintained a significant effect on eribulin PFS in multivariate analysis. Likewise, PFS to first line treatment was positively related to a longer PFS on eribulin treatment, both when it was considered as a continuous variable or as a categorical variable with a 10 months cut-off value (Suppl Table S4).

Survival curves for OS only differed when stratifying patients according to PFS duration on first line and ECOG PS at eribulin start (Suppl Fig. 1B and. 2). In more detail, patients whose PFS on first line > 6 months showed a better survival with respect to those whose PFS was ≤ 6 months, with 12-month OS rates being 58.7% vs. 26.8%, and 24-month OS rates being 14.0% vs. 8.9% (p= 0.02, log-rank test), respectively. Similarly, patients with an ECOG PS at eribulin start of 0 had a better OS compared to those with ECOG PS 1-2, with respective OS rates of 63.6% vs. 26.7% at 12 months, and 27.3% vs. 0.0% at 24 months (p= 0.003, log-rank test). In the univariate model (Suppl Table S5), the variables associated with a lower risk of death from eribulin start were the following: age, longer PFS on first line, PFS on first line > 6 months, and a better ECOG PS at eribulin start. However, in multivariate analysis, only a longer PFS on first line remained as an independent predictor of better OS from eribulin start. Values on pre-and post eribulin ECOG PS were available for 41 patients. Its variation following eribulin treatment (Table 2) resulted statistically significant by using Wilcoxon test (p= 0.0001).

Eribulin administration was generally safe, and no toxic deaths occurred. Two patients (4.5%) discontinued treatment for gastrointestinal toxicity and neurotoxicity, respectively. The most frequent toxicities were fatigue (all grades, 33 patients, 77.3%) and peripheral neuropathy (all grades, 22; 50.0%) (Table 3). Grade 3/4 adverse events (AEs) were recorded in 7 patients (15.9%) and were mostly related to fatigue (3 patients, 6.8%) and neutropenia (2 patients, 4.5%).

In the whole cohort, 26 patients (59.1%) received a third line of treatment, which was more often represented by nab-paclitaxel or a gemcitabine-based regimen (Table 1).

## Discussion

The present study mainly reports on the efficacy of eribulin administered as second-line therapy in mTNBC patients. Overall, eribulin treatment in our cohort was safe, with manageable toxicities. To our knowledge, evidence from literature concerning eribulin use in mTNBC as second-line treatment is lacking, with the majority of data being from retrospective studies 14-19. In the pooled analysis of the EMBRACE trial and Study 301, the PFS on eribulin from TNBC patients was 2.8 months, but most of these patients had received more than 1 previous chemotherapy line 12. Overall, our findings are consistent with results from randomized clinical trials (RCTs), thus confirming the activity of eribulin in the mTNBC subset even in a non-selected patient population.

This study has some limitations. In first place, the topic of interest was addressed using a retrospective design. Heterogeneity in the study population characteristics may represent a further focus of discussion. Further critiques may be fueled by the study sample size, which appears somewhat limited. This may be at least partly explained by the relatively low representation of TNBC among the molecularly defined breast cancer subtypes.

Our study also has some relevant strengths. The topic of choice is extremely timely and of vivid interest to a cancer research agenda. Indeed, TNBC patients represent the breast cancer subgroup with the highest need for innovative treatment options, as witnessed by the recent efforts of the scientific community aimed at broadening the available therapeutic armamentarium. The critical interpretation of the data herein presented, notwithstanding the limitations stemming from the retrospective approach, adds a block of evidence to current knowledge from the pivotal eribulin RCTs and other prior observational studies. In fact, to the best of our knowledge, in none of the prior studies of eribulin in mTNBC, independently on the study design, the authors have specifically focused on the exclusive use of this agent in second line.

In conclusion, our study confirms the role of eribulin as an efficacious and safe option of treatment in the landscape of mTNBC, and an early inclusion in the continuum of treatment strategies could increase the inherent clinical benefits.

## Supplementary Material

Supplementary figures and tables.

## Figures and Tables

**Figure 3:**
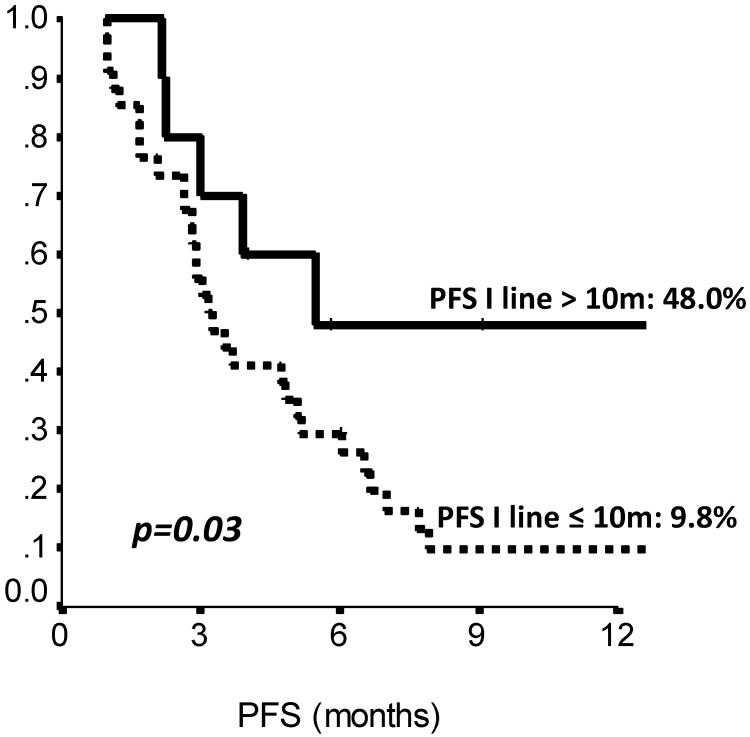
Second line (Eribulin) PFS by length of first line PFS (≤10 vs >10 months). PFS: Progression Free Survival, m: months. N: 44 patients.

**Table 1:** Baseline characteristics of the TETRIS participants (N:44)

Patients' Characteristics	N (%)
Age in years, median (range)	51 (35-81)
**Menopausal status at diagnosis**	
Premenopausal	17 (38.6)
Postmenopausal	27 (61.4)
**Surgery on primary tumor**	
Yes	23 (52.3)
No	21 (47.7)
**Histotype**	
Ductal carcinoma	42 (95.5)
Lobular carcinoma	1 (2.3)
Other	1 (2.3)
**Triple-negative cancer at diagnosis**	
Yes	40 (90.9)
No	4 (9.1)
**Tumor grade**	
2	3 (6.8)
3	40 (90.9)
Missing	1 (2.3)
**BRCA 1/2 mutation**	
Yes	1 (2.3)
No	26 (59.1)
Unknown	17 (38.6)
**Neoadjuvant chemotherapy**	10 (22.7)
(Neo)adjuvant Carboplatin	2 (4.5)
Adjuvant chemotherapy	25 (56.8)
Adjuvant Capecitabine	3 (6.8)
**Metastasis at diagnosis**	
Yes	5 (11.4)
No	39 (88.6)
**Number of metastatic sites**	
1	13 (29.5)
2	20 (45.5)
>2	11 (25.0)
**Pattern of metastatic involvement**	
Visceral	22 (50.0)
Bone-only	2 (4.5)
Other	20 (45.5)
**First-line therapy**	
Paclitaxel	3 (6.8)
Paclitaxel/Bevacizumab	14 (31.8)
Anthracyclines	2 (4.5)
Capecitabine	3 (6.8)
Platinum Salts	11 (25)
Other	11 (25)
**Pretreatment (neo-adjuvant plus first-line)**	
Taxanes	38 (86.4)
Anthracyclines	27 (61.4)
Carboplatin	14 (31.8)
Capecitabine	6 (13.6)
Two agents	19 (43.2)
Three agents	12 (27.3)
**ECOG PS at Eribulin start**	
0	17 (38.6)
1	24 (54.5)
2	2 (4.5)
Unknown	1 (2.3)
**Third line treatment**	
None	18 (40.9)
Nab-paclitaxel	11 (25)
Gemcitabine + Vinorelbine	3 (6.8)
Carboplatin + Gemcitabine	3 (6.8)
Carboplatin	1 (2.3)
Gemcitabine	1 (2.3)
Capecitabine	3 (6.8)
Other	4 (9.1)
**Fourth line treatment**	
Platinum (Cis/Carbo)	2 (4.5)
Capecitabine	2 (4.5)
Anthracyclines	1 (2.3)
Other	1 (2.3)

**Table 2:** ECOG PS at eribulin start vs eribulin end. The TETRIS study (N:44)

ECOG PS at eribulin start	ECOG PS at eribulin end					Total
	0	1	2	3	4	
ECOG PS 0	6	8	2			*16*
ECOG PS 1		10	9	4		*23*
ECOG PS 2			1		1	*2*
Total	*6*	*18*	*12*	*4*	*1*	*41*

*Wilcoxon test: p<0.0001; ECOG PS: Eastern cooperative oncology group performance status.

**Table 3:** Toxicity profile of the TETRIS study participants (N:44)

	All grades, N (%)	Grade 3, N (%)	Grade 4, N (%)
**Hematological**			
Neutropenia	20 (45.5)	2 (4.5)	0
Anemia	18 (40.9)	1 (2.3)	0
Thrombocytopenia	6 (13.6)	1 (2.3)	1 (2.3)
**Non-hematological**			
Fatigue	34 (77.3)	3 (6.8)	0
Peripheralneuropathy	22 (50)	1 (2.3)	0
Nausea/vomiting	20 (45.5)	0	0
Diarrhea	5 (11.4)	0	0
AST/ALT alterations	9 (20.4)	0	0
