# Supplementary material for: Second-line Eribulin in Triple Negative Metastatic Breast Cancer patients. Multicentre Retrospective Study: The TETRIS Trial
Source: Int J Med Sci. 2021 Mar 27;18(10):2245–50. doi: 10.7150/ijms.54996 (PMC8040412; doi:10.7150/ijms.54996)

Supplementary Table 1. First-line survival estimates from the TETRIS study (N:44)

Survival estimates	Median [95%CI] (months)	%
1st-line progression-free survival	7 [1 – 21]	-
Overall survival from first-line	27 [22.4 – 31.6]	-
2-year overall survival from 1st-line	-	57.1

Supplementary Table 2. Second line survival estimates from the TETRIS study (N:44)

Survival estimates	Median, [95%CI] (months)	%
Progression-free survival	3.5 [1.7 – 5.3]	-
1-year Progression-free survival	-	16.7
Overall survival from Eribulin start	11.9 [8.4 – 15.5]	-
1-year Overall survival	-	43
2-year Overall survival	-	12.9

Supplementary Table 3. Univariate analysis of the impact of clinical-pathological features on Progression-free Survival. The TETRIS study (N:44).

	Hazard Ratio (95%CI)	*p* value
Age at eribulin start (years)	0.97 (0.94 - 1.00)	0.063
Menopausal status (yes vs no)	1.02 (0.52 - 2.00)	0.933
Ki-67% (continuous)	1.01 (0.99 - 1.02)	0.090
Ki-67% (>20% vs ≤ 20%)	1.42 (0.55 - 3.71)	0.463
Metastases at diagnosis (yes vs no)	1.30 (0.46 - 3.71)	0.614
Neoadjuvant chemotherapy (yes vs no)	2.14 (1.00 - 4.55)	0.049*
Adjuvant chemotherapy (yes vs no)	0.44 (0.22 - 0.88)	0.020*
Neoadjuvant and/or adjuvant chemotherapy (yes vs no)	1.64 (0.79 - 3.38)	0.178
Visceral disease (yes vs no)	1.06 (0.55 - 2.03)	0.850
First-line progression-free survival	0.91 (0.85 - 0.98)	0.022*

ECOG PS	1.40(0.70 - 2.79)	0.337
BMI (continous)	0.98 (0.92 - 1.06)	0.757
Metastatic sites (3 vs1 - 2)	1.26 (0.59 - 2.70)	0.540
Previous taxanes, any setting (yes vs no)	1.21 (0.46 - 3.14)	0.691

*Statistically significant at an *alpha*<0.05

Legend:

ECOG PS: Eastern Cooperative Oncology Group Performance Status

BMI: Body Mass Index

Supplementary Table 4. Multivariate analysis of factors impacting Progression-free Survival. The TETRIS study (N:44).

	Hazard Ratio (95%CI)	*p* value
Adjuvant chemotherapy (yes vs no)	2.44(1.18 - 5.05)	0.016*
First-line progression-free survival (continuous)	0.92 (0.85 -0.98)	0.018*
First-line progression-free survival (≥10months. vs<10months.)	0.34 (0.13 – 0.89)	0.028*

*Statistically significant at an *alpha*<0.05

Supplementary Table 5. Univariate analysis of factors impacting Overall Survival. The TETRIS study (N:44).

	Hazard Ratio (95%CI)	*p* value
Age at diagnosis (years)	0.97 (0.93 - 1.00)	0.128
Age at Eribulin start (years)	0.96 (0.92 - 1.00)	0.047*
Menopausal status (yes vs no)	1.48(0.62 - 3.53)	0.376
Ki-67% (continuous)	0.99 (0.97 - 1.01)	0.618
Ki-67% (>20% vs ≤ 20%)	1.06 (0.36 – 3.12	0.918
Metastases at diagnosis (yes vs no)	1.32 (0.37 - 4.69)	0.660
Neoadjuvant chemotherapy (yes vs no)	1.99 (0.70 - 5.66)	0.193
Adjuvant chemotherapy (yes vs no)	1.95 (0.85 - 4.41)	0.112
Neoadjuvant + adjuvant chemotherapy (yes vs no)	1.28(0.54 - 3.02)	0.563
Visceral disease (yes vs no)	1.12(0.51 - 2.44)	0.766
Metastatic sites(1 vs 2-3)	1.52 (0.52 – 4.44)	0.442
First-line taxanes	1.45 (0.62 - 3.35)	0.385

First-line progression-free survival	0.87(0.77 –0.98)	0.029*
First-line progression-free survival (> 6mo. vs ≤ 6 mo.)	0.45 (0.20–0.99)	0.046*
ECOG PS	0.20 (0.10 – 0.66)	0.005*
BMI (continuous)	0.96(0.89 - 1.04)	0.354
Previous taxanes, any setting (yes vs no)	1.33 (0.45 - 3.94)	0.599

*Statistically significant at an *alpha*<0.05

ECOG PS: Eastern Cooperative Oncology Group Performance Status

BMI: Body Mass Index

Supplement Figure 1

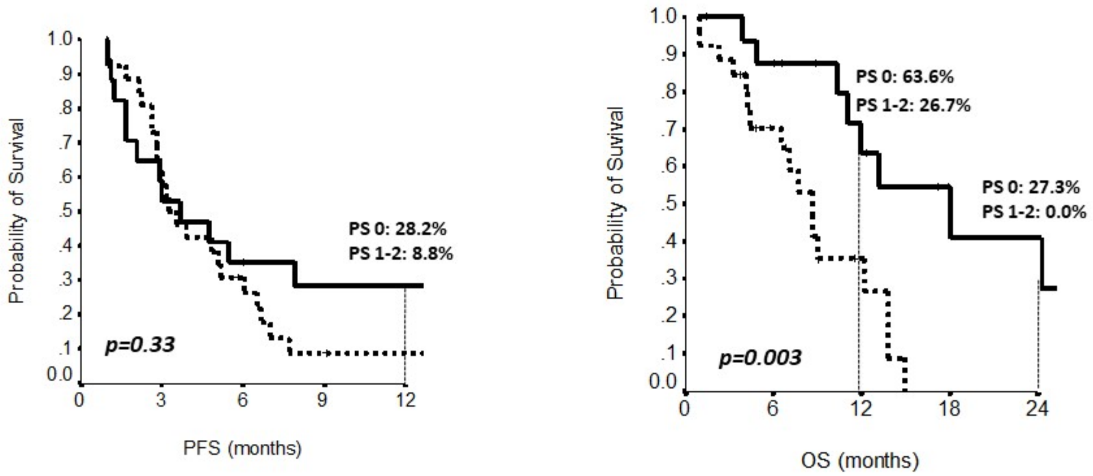

A B

Supplement Figure 2

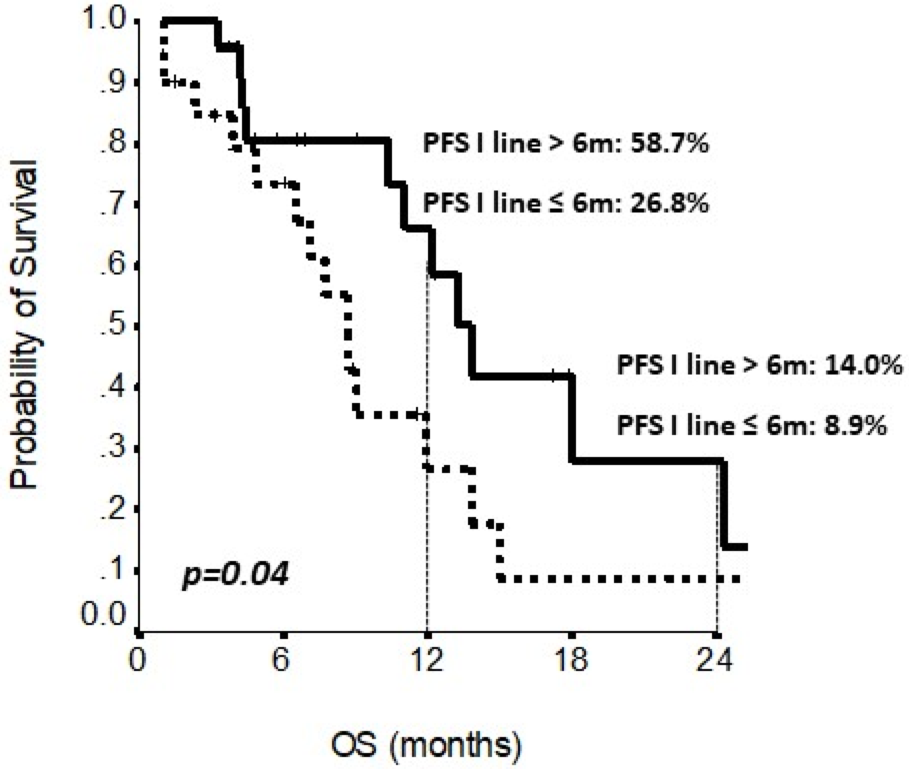

Supplement: Supplementary file 1 — Supplementary figures and tables. [file ijmsv18p2245s1.pdf]
